# Development of acute pancreatitis caused by sodium valproate in a patient with bipolar disorder on hemodialysis for chronic renal failure: a case report

**DOI:** 10.1186/1471-244X-14-93

**Published:** 2014-03-29

**Authors:** Hiroaki Okayasu, Takahiro Shinozaki, Akira Osone, Yuji Ozeki, Kazutaka Shimoda

**Affiliations:** 1 Department of Psychiatry, Dokkyo Medical University, Mibu, Tochigi, Japan

**Keywords:** Acute pancreatitis, Sodium valproate, Chronic renal failure, Hemodialysis, Bipolar disorder, Mood stabilizer

## Abstract

**Background:**

Cases of acute pancreatitis caused by sodium valproate (VPA) have been reported by many authors thus far. However, most of these were cases with epilepsy. Chronic renal failure is also regarded as a risk factor for acute pancreatitis. Here, we report a case of acute pancreatitis development due to VPA in a patient with bipolar disorder on hemodialysis for chronic renal failure.

**Case presentation:**

The patient was a 52-year-old Japanese male who was diagnosed as bipolar disorder on hemodialysis for renal failure. He was treated with VPA and manic symptoms gradually stabilized. However, the patient complained of severe abdominal pain. Blood amylase was found to be markedly high, and computed tomography revealed pancreatomegaly and an increased amount of peripancreatic fat. Hence, we diagnosed the case as acute pancreatitis caused by VPA. We discontinued oral medication, and he was started on a pancreatic enzyme inhibitor, antibiotics, and transfusion, and he showed improvement.

**Conclusion:**

It has been reported that acute pancreatitis induced by VPA is caused by intermediate metabolites of VPA. We consider that patients with renal failure are prone to pancreatitis caused by VPA because of the accumulation of these intermediate metabolites. We need close monitoring for serious adverse effects such as pancreatitis when we prescribe VPA to patients with bipolar disorder on hemodialysis for chronic renal failure, although VPA is safer than other mood stabilizers.

## Background

Acute pancreatitis is a rare, but serious and sometimes fatal, side effect of sodium valproate (VPA) administration. Hemodialysis for the treatment of chronic renal failure is also regarded as a risk factor for acute pancreatitis [1]. Here, we report our experience of a patient with bipolar disorder who developed acute pancreatitis caused by VPA administration while undergoing hemodialysis.

## Case presentation

The patient was a 52-year-old man who had proteinuria and hematuria that were diagnosed in his high-school days but for which he had not sought treatment. He subsequently experienced severe renal failure due to nephrosclerosis and was started on hemodialysis 2 years before this case report. Thereafter, he had a depressive mood, anxiety, and insomnia, and he subsequently presented with symptoms such as pressure of speech, flight of ideas, delusions of grandeur, thought dispersion, and irritability. Diagnosis was bipolar disorder according to DSM-IV-TR diagnostic criteria, and he was treated with olanzapine but showed no improvement. He was admitted to the psychiatric ward of our university hospital.

Because the patient continued to show a threatening attitude and irritability toward medical staff or other patients while being hospitalized, he was initially treated with VPA (400 mg/day) in addition to olanzapine (10 mg/day) and zopiclone (5 mg/day) and other drugs for general medical conditions [nifedipine(40 mg/day), artinolol hydrochloride (20 mg/day), valsartan (160 mg/day) and doxazosin mesilate (3 mg/day) for hypertension, and sodium polystyrene sulfonate (25 g/day) for hyperpotassemia, allopurinol (100 mg/day) for hyperuricemia and alfacalcidol (0.25 μg/day) for chronic renal failure] by the previous physician in charge. The dosage of VPA was increased up to 1,200 mg/day, and the manic symptoms gradually stabilized. The plasma level of VPA was 88.5 μg/ml.

**Figure 1:**
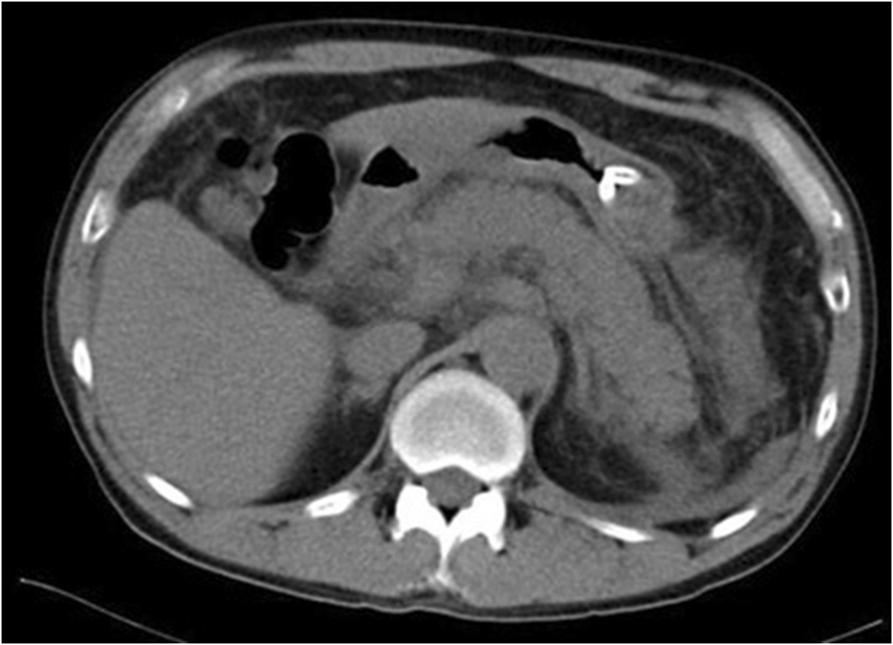
Abdominal computed tomography showing pancreatomegaly and an increased amount of peripancreatic fat.

**Figure 2:**
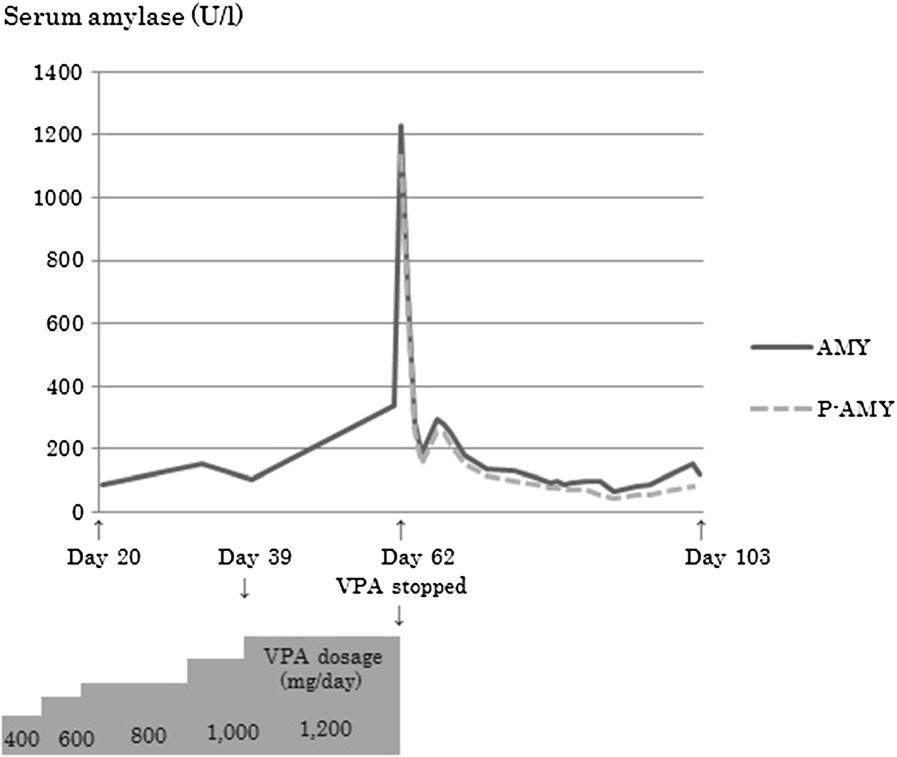
**The course of treatment with VPA and changes in serum amylase.** Abbreviations: AMY, amylase; P-AMY, pancreatic-type amylase; VPA, sodium valproate.

However, approximately 3 weeks after increasing the dose of VPA to 1,200 mg (62 days after admission), the patient complained of severe abdominal pain. Blood amylase was found to be markedly high at 1,227 U/l (pancreatic amylase: 1,133 U/l) (Table 1), and abdominal computed tomography revealed pancreatomegaly and an increased amount of peripancreatic fat (Figure 1). Hence, acute pancreatitis was diagnosed. We believe that the pancreatitis was due to VPA because olanzapine, zopiclone and other drugs for general medical conditions had shown no particular adverse effects, and the patient had not consumed alcohol and did not have a history of biliary system disease such as gallstones. We promptly discontinued oral medication, and he was started on a pancreatic enzyme inhibitor, antibiotics, and transfusion, and he showed improvement for approximately 1 month (Figure 2). Because the patient’s psychiatric symptoms were exacerbated, levomepromazine (50 mg/day) was then started without any adverse effects. He was discharged on Day 197 of the illness.

**Table 1:** Laboratory data at the time when acute pancreatitis was diagnosed

**Laboratory parameters**	**Patient’s values**	**Laboratory parameters**	**Patient’s values**
AST	17 U/l	Glu	109 mg/dl
ALT	5 U/l	CRP	**7.59 mg/dl**
ALP	171 U/l	Lipase	**959 U/l**
γ-GTP	20 U/l	WBC	**17,700/mm**^**3**^
T-Bil	0.3 mg/dl	NEUTRO	**82.7%**
BUN	**53 mg/dl**	EOSINO	0.0%
Cre	**10.52 mg/dl**	BASO	0.1%
Na	138 mEq/l	MoC	8.8%
K	4.7 mEq/l	LYMPHO	8.4%
Cl	102 mEq/l	RBC	373 × 10^4^/mm^3^
CK	72 U/l	Hb	11.4 g/dl
AMY	**1,227 U/l**	Ht	35.8%
P-AMY	**1,133 U/l**	Plt	11.9 × 10^4^/mm^3^

*Abbreviations*: AST, aspartate transaminase; ALT, alanine transaminase; ALP, alkaline phosphatase; γ-GTP, γ-glutamyl transpeptidase; T-Bil, total bilirubin; BUN, blood urea nitrogen; Cre, creatinine; Na, sodium; K, potassium; Cl, chloride; CK, creatine kinase; AMY, amylase; P-AMY, pancreatic-type amylase; Glu, glucose; CRP, C-reactive protein; WBC, white blood cells; NEUTRO, neutrophil; EOSINO, eosinophil; BASO, basophil; Moc, monocyte; LYMPHO, lymphocyte; RBC, red blood cells; Hb, hemoglobin; Ht, hematocrit; Plt, platelets.

Numbers in bold type indicate abnormal value.

### Discussion

In 1979, Canfield et al. [2] and Batalden et al. [3] separately reported cases of acute pancreatitis caused by VPA. Since then, more than 100 such cases have appeared in the literature [4], although most cases involved epilepsy, and only two cases involved bipolar disorder [5,6]. Ng et al. reported a female patient with epilepsy who was administered VPA and developed pancreatitis during hemodialysis [7]. Since then, the similar types of cases that have been reported thus far were cases with epilepsy [1,8-10]. To the best of our knowledge, the present report is the first case of VPA-induced pancreatitis in a patient with bipolar disorder who was also undergoing hemodialysis.

Torelli et al. reported that the intermediate metabolites of VPA have toxic effects on pancreatic cells, and this is believed to be the main cause of VPA-induced pancreatitis [11], although the definite metabolite has not been revealed yet. VPA is primarily metabolized as a glucuronic acid-conjugated compound [10]; however, several types of intermediate metabolites other than the glucuronic acid-conjugated compound are found in normal human urine. One possible etiology is that patients with renal failure are prone to pancreatitis because these intermediate metabolites accumulate due to renal failure leading to pancreatic cell damage.

When considering regimens of other mood stabilizers for patients with bipolar disorder who have renal failure, lithium carbonate does not bind to the plasma protein, and more than 95% of lithium is excreted by the kidney [12]. Therefore, patients with renal dysfunction would be expected to accumulate lithium and show a decline in renal function and serious lithium toxicity. In addition, maintaining adequate blood levels of lithium to achieve a response may be difficult because of the high dialyzability of lithium [13]. For administration of carbamazepine (CBZ), close monitoring of patients with renal failure is believed to be required because toxicity can readily occur as a result of accumulation of toxic CBZ-hydroxylated metabolites due to low albuminemia and inhibition of CBZ binding to the serum protein by a uremic toxin [14]. Therefore, using other mood stabilizers such as these for patients on hemodialysis requires close therapeutic drug monitoring. Physicians should be alert to signs of serious toxicity and the onset of adverse effects.

VPA is primarily metabolized by the liver; therefore, the management of the dose and blood level should be relatively easy because of its low dialyzability [15]. Therefore, VPA seems to be safe even for patients on hemodialysis. However, as in the present case, we must be cognizant of the possibility of serious adverse effects such as pancreatitis.

## Conclusion

When prescribing a mood stabilizer to patients with bipolar disorder on hemodialysis, VPA is believed to be safer than other mood stabilizers. However, it is necessary to monitor patients closely for serious adverse effects such as pancreatitis.

## Consent

The patient has given written consent for publication of this case report.

## Competing interests

The authors declare that they have no competing interests.

## Authors’ contributions

HO produced the initial draft including interpretation of the case findings. TS, AO, YO and KS critically revised the draft. All authors have read and given final approval to the version to be published.

## Pre-publication history

The pre-publication history for this paper can be accessed here:

http://www.biomedcentral.com/1471-244X/14/93/prepub
